# Effect of Leventhal’s self-regulatory intervention on the hypertensive patients’ illness perception and lifestyle: a randomized controlled trial

**DOI:** 10.1186/s12872-023-03049-6

**Published:** 2023-01-26

**Authors:** Fatemeh Saranjam, Ardashir Afrasiabifar, Alikaram Alamdari, Nazafarin Hosseini

**Affiliations:** 1 Student Research Committee, Yasuj University of Medical Sciences, Yasuj, Iran; 2 Professor of Nursing, Yasuj University of Medical Sciences, Yasuj, Iran; 3 Nursing Department, Nursing School, Yasuj University of Medical Sciences, Yasuj, Iran; 4 Social Determinants of Health Research Center, Yasuj University of Medical Sciences, Yasuj, Iran; 5 School of Nursing, Yasuj University of Medical Sciences, Yasuj, Iran

**Keywords:** Self-regulatory model, Illness perception, Hypertension, Lifestyle

## Abstract

**Background:**

The perception of illness may lead to improving the hypertensive patients’ lifestyle, but no study was found in this regard. Therefore, this study was conducted to determine the effect of intervention based on Leventhal’s self-regulatory model on the illness perception and lifestyle of patients with hypertension.

**Methods:**

In the present randomized controlled trial study, ninety eligible patients with primary hypertension were randomly assigned to one of the two groups of intervention and control. Patients in the intervention group received five sessions of Leventhal’s self-regulatory intervention, each of 60 min and weekly. However the control group received routine care. The outcomes were illness perception and lifestyle of the patients with hypertension. The Revised Illness Perception Questionnaire and the Lifestyle Questionnaire were administered to assess illness perception and lifestyle before the treatment to establish a baseline and subsequently 12 weeks after the intervention. The collected data were analyzed using statistical IMB SPSS software, version 21. Qualitative data were analyzed using Chi-Square test or Fisher’s Exact test, and the Independent Sample t- test and Paired Sample t- test were used for analyzing quantitative data.

**Results:**

Leventhal’s self-regulatory intervention improved subscales of illness perception (*p* < 0.05) except for emotional representations and consequences. The global mean scores of the hypertensive patients’ lifestyle in the intervention group significantly increased from 102.8 ± 2.3 at the baseline to 112.1 ± 3 post-intervention.

**Conclusions:**

Interventions based on Leventhal’s self-regulatory model could improve the illness perception and lifestyle of patients with hypertension.

*Trial registration* The present randomized controlled trial study was registered on the Iranian Registry of Clinical Trials Website (IRCT); ID: IRCT20141222020401N6 on 8/5/2019.

## Introduction

Hypertension (HTN) is a significant risk factor for the cardiovascular disease, which is a leading cause of mortality [1]. In people 18 years and older, blood pressure less than 80.120 mmHg is normal blood pressure, systolic pressure 120–139 mmHg, or diastolic pressure 80–89 mmHg is prehypertension stage. Moreover, systolic pressure of 140–159 and diastolic pressure of 90–99 mm Hg is classified as stage 1 hypertension. A systolic pressure ≥ 160 or a diastolic pressure ≥ 100 mm Hg is classified as stage 2 hypertension [2]. HTN is an asymptomatic disease and hence, most patients are unaware of the illness. As a result, they may have already major health problems such as damage to their brain and kidneys once they are diagnosed with HTN [3]. This disease is often called ‘silent killer’ since it has a high mortality rate but no symptoms [4]. Even after diagnosis, many patients do not adhere to the treatment and recommendations needed to control the disease. In addition to medication, maintaining a healthy lifestyle, which involves being physically active, quitting smoking and alcohol, managing stress [5], and following the Dietary Approaches to Stopping Hypertension (DASH), is of great importance in controlling HTN [6]. Illness misperception has negative effects on patients’ behaviors such as adherence to treatment, self-diagnosis, help-seeking behavior and the disease outcomes [7].

Perception of the disease is based on patients' beliefs and perceived knowledge of their condition and can affect their mental health and how they deal with the disease [8]. The illness perception frames living with illness mentally. In illness perception, the coherence of health information affects the cognitive representation and emotional response. For example, positive or negative beliefs about the disease can affect the ability to cope with the disease and perceive it as manageable or threatening, affecting mental health and health behavior such as adherence to treatment [9]. Illness perception (IP) has been described as part of Leventhal’s self-regulatory model [10]. The original model consists of five main subscales: identity, timeline (acute/chronic/cyclical), consequences, cause, and control/cure (treatment control and personal control). Subsequently, the two subscales of emotional representations and illness coherence were added to the model [11]. Leventhal’s self-regulatory model is useful for gaining insights into how people with hypertension think of their illness and how this affects their adherence to,therapeutic regimens, and health outcomes [12]. Patients select and evaluate self-care behaviors based on the manifestations of their illness. For example, someone who perceives hypertension as a chronic disease and views it as a result of lifestyle factors is likely to change their lifestyle first and then seek proper medication interventions. However, suppose a person perceives hypertension acute. In that case, they may not want to change their lifestyle and prioritize receiving medical interventions and adhering to treatment [13].

The expectation is that, through cognitive and emotional responses, IP can increase patients’ motivation to improve their lifestyles [14]. For example, a study by Yan et al. indicated that improved illness perception could affect the lifestyle of patients with myocardial infarction [15]. Moreover, Rakhshan et al. found that IP interventions positively affect the lifestyle of patients with metabolic syndrome [16]. In another study, training based on Leventhal’s self-regulatory model in patients with hypertension improved adherence to treatment and reduced patients’ blood pressure [17]. However, in the study by van Broekhovena et al., more threatening IP was not associated with positive lifestyle changes in gynecological cancer patients [14].

The researchers have not found a study on the effect of IP intervention on the lifestyle of patients with HTN. Therefore, this study was conducted to determine the effect of intervention based on Leventhal’s self-regulatory model on the illness perception and lifestyle of patients with hypertension.

## Material and methods

### Design and participants

The present randomized parallel-controlled trial study was registered on the Iranian Registry of Clinical Trials Website (IRCT), ID: IRCT20141222020401N6, on 8/5/2019. The present study was conducted on ninety eligible hypertensive patients referring to Yasuj Shahid Dastgheib Health Center, Iran, from May 2019 to October 2019. A total of 41 participants were calculated as the sample size for each group considering α = 0.05, z_1-α/2_ = 1.96, β = 0.2, 1-β = 0.8, z_1-β_ = 0.85, lifestyle standard deviations of S_1_ = 26.59 and S_2_ = 16.96, and lifestyle means of μ_1_ = 21.8 and μ_2_ = 41.2 [18], using the following formula:

$$\mathrm{n}=\frac{2\times \left[{\left({\mathrm{z}}_{1-\frac{\mathrm{\alpha }}{2}}+{\mathrm{z}}_{1-\upbeta }\right)}^{2}\right]\times \left({\mathrm{S}}_{1}^{2}+{\mathrm{S}}_{2}^{2}\right)}{{\mathrm{d}}^{2}={\left({\upmu }_{1}-{\upmu }_{2}\right)}^{2}}$$

According to the researcher's guess and dropout prediction in interventional studies, 10% attrition rate was considered. Therefore, a total of 90 participants, each group comprising 45 patients, participated in the present study.

Patients with hypertension who had health records in Shahid Dasghib Health Center were selected as research participants. The telephone numbers of the patients were contacted, and the objectives of the research were explained to them. Those who wanted to participate in this research were invited to attend the health center and were assessed in terms of inclusion and exclusion criteria. Then, written informed consent was signed by eligible patients. The written informed consent included familiarizing with the research, goals, and interventions, stating the advantages and disadvantages of participating in the research and compensating for the disadvantages, maintaining the confidentiality, and the right to withdraw from the study.

They were selected through convenience sampling. However, they were randomly assigned to one of the two groups of intervention (n = 45) and control (n = 45), using the randomized block allocation method as follows: Initially, by multiplying the number of study groups by two (an intervention group and a control group), four people were assigned to each group. At that point, twenty-four blocks were calculated using the factorial rule (24 = 1 × 2 × 3 × 4 = !4). The members of each block were marked with the letters A, B, C, D. Subsequently, letters A and B were assigned to the control group, and letters C and D were randomly assigned to the intervention group. A total of twenty-four blocks with possible layouts were identified. Allocation was done by randomly selecting each block by an individual outside the research team.

Furthermore, the samples were selected based on the sequence of blocks and the time priority of the participants' entry. Randomization continued until 45 patients were in the intervention group and 45 in the control group. Blinding was not done in the present study.

Inclusion criteria consisted of a definitive diagnosis of primary hypertension, stage 1 or 2 hypertension, age range of 18–65 years, at least six months of hypertension, informed consent to participate in the study, and lifestyle score of ≤ 105. Patients’ unwillingness to participate in the study, not having other chronic diseases or severe complications following hypertension and lack of inclusion criteria were considered exclusion criteria.

### Instrument and data gathering

The outcomes were Lifestyle and Illness Perception, the formerly measured by the Lifestyle Questionnaire (LSQ) and the latter by the Revised Illness Perception Questionnaire (IPQ-R) two times: the baseline (week 0) and 12 weeks following the intervention (week 17). The IPQ-R was originally developed by Moss-Morris et al. [19] to assess patients̓ illness perception. This questionnaire comprises 70 items which are divided into nine subscales: identity (attributing unrealistic symptoms to the disease), consequences (belief in negative consequences of the disease), timeline acute/chronic (patients’ perception of the illness chronicity), timeline cyclical (believing that the disease is cyclical until its stability), personal control (belief in more control), treatment control (belief in more treatment), emotional representations, illness coherence (higher level of illness perception), and perception of the causes. The score of the identity subscale was obtained by adding up the positive answers to symptoms. Furthermore, the subscales of 2 to 9 were based on a 5-point Likert scale (strongly disagree: 1, disagree: 2, neither agree nor disagree: 3, agree: 4, and strongly agree: 5). A lower score in the subscales of identity, consequence, timeline cyclical and emotional representations indicates a higher perception. On the contrary, higher scores in the subscales of timeline acute/chronic, personal control, treatment control, and illness coherence indicate a higher perception of the disease.

The validity and reliability of the IPQ-R had previously been confirmed. The Cronbach alpha’sfor each of the subscales ranged from 0.79 for the timeline cyclical dimension to 0.89 for the timeline acute/chronic dimension. The average scale content validity (S-CVI_Ave_) for each of the dimensions was as follows: Consequences was 0.75, Timeline acute/chronic was 0.75, Treatment control was 0.89, Personal control was 0.81, Emotional representations was 0.77, Illness coherence was 0.74, and Timeline cyclical was 0.66. The S-CVI_Ave_ for the whole questionnaire was 0.79 [19, 20]. The validity and reliability of the Persian version of the questionnaire had likewise been established. The internal consistency of the scales was over 0.78 [21].

Lifestyle was assessed by LSQ. The LSQ consists of 70 items divided into 10 subscales: physical health, exercise, and fitness; weight control and nutrition; illness prevention; psychological health; spiritual health; social health, avoidance of drugs, opiates, and alcohol, prevention of accidents and environmental health. The LSQ score is based on a four-point Likert scale (never = 0, sometimes = 1, usually = 2, and always = 3). Global score of the LSQ ranges from zero to 210. The validity of LSQ had been established through content validity by 10 experts, factor analysis (10 factor with a factor load of 0.31 to 0.88 and an specific value of 1.04 to 6.23), and convergence validity (r = 0.59–0.62). Cronbach's alpha (r = 0.76–0.89) and test–retest (r = 0.84–0.94) were used to determine reliability of the questionnaire. Cronbach’s alpha for the whole questionnaire was 0.87 [22].

### Interventions

The intervention protocol designed based on Leventhal’s self-regulatory model and the literature review focusing on the subscales of illness perception and lifestyle [16, 23]. Due to a large number of patients in the intervention group, the patients were divided into three groups. The intervention, on a weekly basis (5 weeks), five 60-min sessions were held for the intervention group. The intervention was performed by one of the researchers (master’s student in Community Health Nursing), a psychologist, and a nutritionist consistentin Shahid Dastgheib Health Center as following:.

In the first session, the purpose was to increase the patients' perception of illness identity and causes, especially those related to lifestyle. For this purpose, the patients’ perception of the illness identity was discussed by asking several questions about the symptoms, the cause (s) of the disease, and lifestyle factors believed to have contributed to the disease. In this session, the patient's perception of the illness identity and causes was determined, and the pathophysiology, causes, and symptoms of hypertension were discussed.

In the second session, the purpose was to increase the perception of the patients about the effect of hypertension on their life and the disease consequences, the disease duration, personal control, and treatment control. The patients were evaluated by asking the following open-ended questions: How long do you think it will take to recover? Do you think your disease can be controlled and cured? What will be the consequences of this disease for you? Moreover, misconceptions of the relevant issues were clarified through discussion between the patients and the researcher. The use of drugs and their side effects were also discussed.

In the third session, the purpose was to improve the patients' perception about the subscales including the illness coherence and the necessity of avoiding drugs, opiates, and alcohol, emotional representations, as well as psychological, spiritual, and social health. The patients and the researcher discussed illness coherence and the necessity of avoidance of drugs, opiates, and alcohol. Following that, the psychologist talked about emotional representations and psychological, spiritual, and social health, using counseling techniques and providing the necessary training.

In the fourth session, the purpose was to increase the patients' perception about the subscales such as Weight control, nutrition, and physical health. To this, the participants were asked about their perception of and adherence to weight control and nutrition in hypertension and were advised about proper diet. Moreover, the researcher discussed the importance of maintaining physical health.

In the fifth session, the purpose was to improve the patients' perception of exercise and fitness, environmental health, and prevention of accidents and illness. Therefore, this session was devoted to the patient's perception of the importance and benefits of exercise and fitness, environmental health, and prevention of accidents and illness. Meanwhile, proper educational interventions were provided. At the end of each session, an educational pamphlet containing a summary of the educational content was handed over to the patients.

However, the control group received routine education based on the hypertensive guideline, face-to-face in the health centers.

The data were collected before the intervention as the baseline (week 0) and 12 weeks after the intervention (week 17) [24, 25].

### Data analysis

The data were analyzed, using inferential statistics. The nominal data were analyzed by Chi-Square test or Fisher’s Exact test. For quantitative data with normal distribution, independent sample t-test and paired t-test were used. *P*- value < 0.05 was considered a significant difference for all data analyses. The data analyzer was blind to the allocation of the patients to the groups.

## Results

**Fig. 1:**
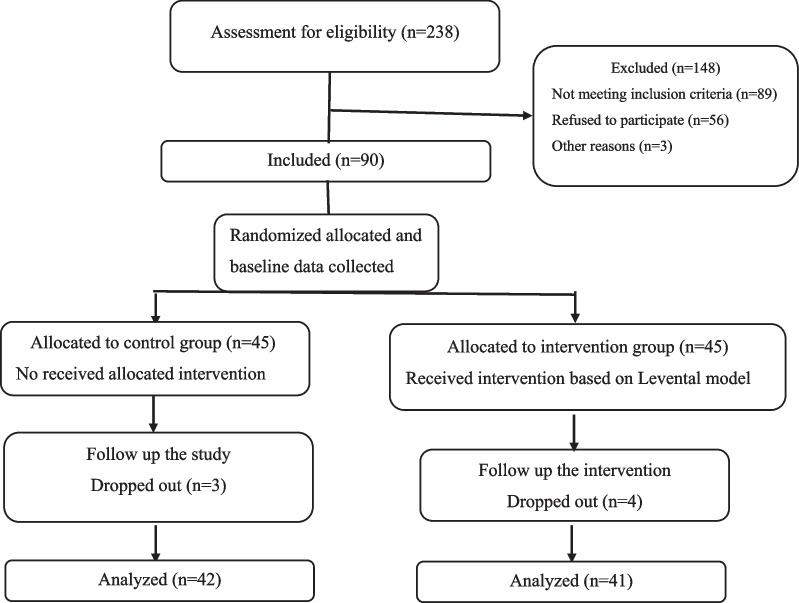
CONSORT flowchart of the study

Ninety hypertensive patients initially consented to participate in the present study. However, seven patients either withdrew or failed to complete the intervention (Fig. 1). The mean value of the participant's age was 53 ± 6.5 years (Range 37–65). All hypertensive patients were married and taking oral antihypertensive drugs at the time of the study. Moreover, most of them were female (84.3%), housewives (79.5%), and had undergraduate education (79.5%). In terms of demographic variables and disease characteristics, including duration of hypertension, there was no significant difference between the participants in the intervention and control groups before the intervention (Table 1).Likewise, before the intervention, there was no statistically significant difference between the intervention and control groups in terms of subscales of illness perception. However, after the intervention, the scores of subscales of illness perception improved significantly (*p* < 0.05), compared with the hypertensive patients in the control group except for illness consequences (*p* = 0.1) and emotional representation subscales (*p* = 0.07) (Table 2).

**Table 1:** Comparing demographic characteristics between the two groups

Characteristics		Group		
		Control N = 42	Intervention N = 41	*P*- value^a^
M ± SD	Age	55.1 ± 6	52.5 ± 6.8	0.07
Gender; N (%)	Male	9(21.4)	4(9.8)	0.1
	Female	33(77.6)	37(90.2)	
Education; N (%)	Under Diploma	32(76.2)	34(82.9)	0.3
	Diploma	10(23.8)	7(17.1)	
Job; N (%)	Home maker	30(71.4)	36(87.8)	0.4
	Others	12(28.6)	5(12.2)	
Family history; N (%)	Yes	31(73.8)	30(73.2)	0.5
	No	11(26.2)	11(26.8)	
Personal history (month)	M ± SD	88.5 ± 66.6	69.2 ± 44.4	0.1

*M ± SD* Mean ± Standard deviation, *N (%)* Frequency (percent)

*P*- value^a^ are based on Independent sample *t*-test for Age variables and Chi- square test for others variables

**Table 2:** Between and within group comparison for illness perception

Subscale	Group time		Intervention	Control	Between group comparison
			Mean ± SD (95% CI)	Mean ± SD (95% CI)	*p*-value
Identity	Before		3.5 ± 1.6 (3.1–4.1)	3.9 ± 1.8 (3.4–4.5)	0.3
	Post		2.8 ± 1.5 (2.3–3.2)	3.9 ± 1.9 (3.3–4.5)	0.006
	Within group comparison	Effect size	0.4	0.002	
		*p*-value	0.001	0.7	
Timeline (acute/chronic)	Before		19.1 ± 4.3 (17.7–22.4)	19.9 ± 3.3 (18.9–20.9)	0.3
	Post		21.7 ± 3.4 (20.5–22.7)	19.8 ± 2.9 (19–20.7)	0.009
	Within group comparison	Effect size	0.5	0.002	
		*p*-value	0.001	0.8	
Consequences	Before		19.4 ± 5.6 (17.7–21.2)	18.1 ± 6.2 (16.1–19.9)	0.3
	Post		18.5 ± 4.9( 16.8–20.2)	18.1 ± 5.5(16.3–19.8)	0.7
	Within group comparison	Effect size	0.1	0.001	
		*p*-value	0.001	0.9	
Personal control	Before		21.2 ± 4.2( 19.8–22.6)	20.5 ± 3.3 (19.5–21.5)	0.3
	Post		22.7 ± 3.1( 21.7–23.7)	20.9 ± 3.5 (19.9–22)	0.01
	Within group comparison	Effect size	0.2	0.07	
		*p*-value	0.001	0.08	
Treatment control	Before		20.2 ± 2 (19.5–20.8)	19.9 ± 1.8 (19.4–20.5)	0.4
	Post		21.5 ± 2 (20.9–22.1)	20.1 ± 2 (19.5–20.8)	0.003
	Within group comparison	Effect size	0.4	0.03	
		*p*-value	0.001	0.3	
Illness coherence	Before		15.6 ± 3.1 (14.7–16.6)	15.8 ± 3.6 (14.7–16.9)	0.7
	Post		17.9 ± 2.8 (17.1–18.9)	16.1 ± 3.6( 15.1–17.2)	0.01
	Within group comparison	Effect size	0.5	0.03	
		*p*-value	0.001	0.3	
Timeline cyclical	Before		13.8 ± 2.7 (13–14.7)	13.19 ± 3.1( 12.2–14.1)	0.2
	Post		11.5 ± 2.3 (10.8–12.2)	13 ± 2.7 (12.1–13.8)	0.01
	Within group comparison	Effect size	0.5	0.01	
		*p*-value	0.001	0.5	
Emotional representations	Before		22.9 ± 5.5 (21.3–24.5)	20.7 ± 6.2 (19–22.7)	0.1
	Post		22 ± 4.5 (20.6–23.2)	20.9 ± 5.5 (19.3–22.6)	0.3
	Within group comparison	Effect size	0.08	0.01	
		*p*-value	0.05	0.7	

Between group comparison based on t-test

Within group comparison based on paired t-test

In the intragroup comparison, the results showed a significant improvement in the subscales of illness perception in the intervention group (*p* = 0.001) after the intervention, except for the emotional representation subscale. However, in the control group, no significant difference was observed in any of the subscales after the intervention compared to the time before the intervention (Table 2).

In addition, before the intervention, there was no statistically significant difference between the intervention and control groups in terms of lifestyle and its subscales. However, the results indicated that after the intervention, the hypertensive patients in the intervention group reported significantly (*p* < 0.05) more improvement in their lifestyle and its subscales, compared with the hypertensive patients in the control group, except for psychological health (*p* = 0.6) spiritual health (*p* = 0.5) and social health (*p* = 0.09) (Table 3).

**Table 3:** Between and within group comparison for life style

Subscale	Group time		Intervention	Control	Between group comparison
			Mean ± SD(95% CI)	Mean ± SD(95% CI)	*p*-value
Global life style	Before		102.8 ± 2.3 (102–03.5)	112.1 ± 3 (111.1–13.1)	0.6
	Post		112.1 ± 3 (111.1–113.1)	103.5 ± 2.1 (102.9–104.2)	0.001
	Within group comparison	Effect size	0.8	0.07	
		*p*-value	0.001	0.09	
Physical health	Before		9.1 ± 2.4 (8.4–9.8)	9.2 ± 2.2 (8.4–9.8)	0.7
	Post		10.6 ± 1.8 (10.1–11.2)	9.1 ± 2.2 (8.4–9.7)	0.001
	Within group comparison	Effect size	0.7	0.02	
		*p*-value	0.001	0.4	
Exercise and fitness	Before		7.5 ± 3.8 (6.3–8.7)	7 ± 3.2 (6–7.9)	0.5
	Post		8.9 ± 3.2 (8 -9.9)	6.9 ± 2.5 (6.1–7.7)	0.002
	Within group comparison	Effect size	0.5	0.002	
		*p*-value	0.001	0.8	
Weight control and nutrition	Before		9.1 ± 2.5 (8.4–9.8)	9 ± 2.4 (8.3–9.7)	0.8
	Post		10.8 ± 2.4 (10.1–11.6)	9.1 ± 2.2 (8.5–9.8)	0.002
	Within group comparison	Effect size	0.7	0.006	
		*p*-value	0.001	0.6	
Environmental health	Before		11.3 ± 2.4 (10.5–12)	10.6 ± 1.9 (10–11.3)	0.2
	Post		11.9 ± 2.2(11.2–12.7)	10.7 ± 1.7 (10.1–11.2)	0.005
	Within group comparison	Effect size	0.2	0.001	
		*p*-value	0.001	0.7	
Illness prevention	Before		10.8 ± 1.3 (10.5–11.3)	11.1 ± 1.3 (10.7–11.6)	0.3
	Post		12.5 ± 1.6(11.9–13)	11.3 ± 1.5 (10.9–11.8)	0.002
	Within Group comparison	Effect size	0.5	0.04	
		*p*-value	0.001	0.1	
Psychological health	Before		8.5 ± 2.3 (7.9–9.3)	9.3 ± 2.1( 8.7–9.9)	0.1
	Post		8.9 ± 2.1 (8.3–9.5)	9.1 ± 2 (8.5–9.7)	0.6
	Within group comparison	Effect size	0.08	0.03	
		*p*-value	0.06	0.2	
Spiritual health	Before		10.7 ± 2.1(10.1–11.4)	10.6 ± 2.5 (9.8–11.4)	0.7
	Post		10.6 ± 2.3(10.2–11.6)	10.6 ± 2.5 (9.9–11.4)	0.5
	Within group comparison	Effect size	0.05	0.003	
		*p*-value	0.1	0.7	
Social health	Before		10.5 ± 1.8(9.9–11.1)	11.1 ± 1.9 (10.5–11.2)	0.1
	Post		10.6 ± 1.7(10.1–11.2)	11.3 ± 1.71 (0.8–11.8)	0.09
	Within group comparison	Effect size	0.02	0.03	
		*p*-value	0.3	0.2	
Avoidance of drugs, opiates and alcohol	Before		13.1 ± 1.6 (12.6–13.7)	13.5 ± 1.6 (12.9–13.9)	0.3
	Post		13.9 ± 1.6 (13.5–14.4)	13.7 ± 1.9 (13.1–14.3)	0.01
	Within group comparison	Effect size	0.5	0.07	
		*p*-value	0.001	0.08	
Prevention of accidents	Before		12 ± 2.6 (11.2–12.8)	11.3 ± 2.1 (10.7–12)	0.2
	Post		12.9 ± 2.3 (12.3–13.7)	11.5 ± 1.7 (11–12.1)	0.002
	Within group comparison	Effect size	0.5	0.01	
		*p*-value	0.001	0.4	

Between group comparison based on t-test

Within group compariason based on paired t-test

The results showed that the intervention based on Leventhal’s self-regulatory model caused a significant increase in lifestyle and its subscales in the intervention group (*p* < 0.05) after the intervention, as compared to the time before the intervention, except for psychological, spiritual, and social health. However, in the control group, in terms of the same variables, there was no significant difference before and after the intervention (Table 3).

## Discussion

To the best of our knowledge, no study has been carried out to determine the effect of interventions based on Leventhal’s self-regulatory model on the illness perception and lifestyle of patients with hypertension. Therefore, the present study aimed to fill this lacuna. The results showed that intervention based on the present model improved illness perception in the subscales of the identity, timeline acute/chronic, personal control, treatment control, illness coherence, and timeline cyclical. However, it had no significant effect on the consequences and emotional representations.

As suggested by the present study's findings, the illness perception-based intervention improved the illness coherence subscale in the study of Broadbent et al. (2009) in the spouses of patients with myocardial infarction [26]. In contrast, Cossette et al. indicated that cardiac rehabilitation nursing intervention in patients with the acute coronary syndrome has no effect on the illness coherence subscales and timeline acute/chronic, treatment control, and timeline cyclical subscales [27]. The difference between the results of these two studies indicates that interventions based on improving the illness perception can be more effective in improving illness coherence and other subscales than other educational interventions.

In addition, participants in the present study better perceived their illness identity after the intervention, including the symptoms and the disease timeline, acute/chronic. This is in line with the study's findings by Yan et al. [15]. Among other things, they found that the training program based on Leventhal’s model increases patients’ perception of the symptoms and disease duration after myocardial infarction [15]. In this study, increasing patients’ perception of the chronic timeline of hypertension improved patients’ adherence to treatment and several lifestyle subscales.

The present study's findings confirmed the results obtained by Lee et al. in patients with injury [28] and Weldam et al. [29] in patients with chronic obstructive pulmonary disease. Similarly, Richardson et al. found that the treatment control subscale was promoted after the self-regulatory model-based intervention in cancer patients in the intervention group. However, no significant improvement was observed in the follow-up study with an interval of 6 months [30]. This may be attributed to the frustration and fatigue of cancer patients. It is also necessary to note that in the present study, it was not possible to conduct a follow-up study. Therefore, no meaningful comparison can be made in this regard.

As in the study by Rakhshan et al. [16], in the present study, no significant difference was observed in terms of the subscales of the consequences and emotional representations, even if it has already been reported that emotional representations can affect self-care and health consequences [31]. It can be argued that to improve the perception of consequences, and emotional representations should be improved in patients with hypertension. This probably requires long-term training programs and proper psychological interventions.

The results of the present study indicated that after the intervention, the scores of total lifestyles and its subscales, except for spiritual and social health, increased in the intervention group, as compared with the control group. Likewise, in Yan et al.’s study, an educational program based on Leventhal’s model improved nutrition and physical activity in patients after myocardial infarction [15]. Likewise, the study by Shayesteh et al. revealed that following lifestyle-based intervention**,** the overall score of lifestyle and physical activity increased in patients with hypertension [32]. The study by Dehghani et al. also indicated that lifestyle-based intervention in patients with coronary heart disease reduced obesity and increased physical activity in patients [33]. As in the present study, the intervention affected the weight management of participants by creating a better perception of the disease and a sense of threat.

Blood pressure is affected by environmental factors such as noise and air pollution. Therefore, patients should be informed to avoid exposure to these factors [34]. Familiarizing patients with risk factors and changing their high-risk behaviors are the main objectives of the prevention subscale [35]. In the present study, all of the issues mentioned above were incorporated into the education of the patients, and, as a result, these subscales improved after the intervention. Moreover, the subscale of avoidance of drugs, opiates, and alcohol improved after the interventions. Similar to this study, Dehghani et al. indicated that lifestyle-based intervention in patients with coronary heart disease helped them resist the urge to smoke [33].

In the present study, the psychological health subscale did not improve after the research intervention. Similarly, Rakhshan et al. found that education based on perception in patients with metabolic syndrome did not affect stress management, although it improved all areas of lifestyle [16]. Contrary to the results of the present study, the study by Shayesteh et al. showed that the educational intervention improved stress management in patients with hypertension [32]. Moreover, findings by Sararoudi et al. showed that the interventions based on Leventhal’s self-regulatory model reduced anxiety and depression in patients with myocardial infarction [36]. Patients’ psychological health was expected to improve with increased illness perception. This difference may be due to the different natures of diseases targeted in the above studies. However, in this study, the intervention based on Leventhal’s model did not improve psychological, social, and spiritual health in patients with hypertension. According to these results, other spiritual and behavioral interventions may be needed to improve psychological, social, and spiritual health.

The present study's overall lifestyle score was improved, probably due to increased perception. In their systematic review study, French et al. showed that patients with acute myocardial infarction and a positive perception of identity, consequences, cure/control, and illness coherence feel the need for cardiac rehabilitation [37]. Although the variable measured in the study above differs from the variable of consequences in the present study (i.e., healthy lifestyle), it was shown in both studies that a higher illness perception is associated with the acceptance of health-related behaviors.

In contrast to the results of the present study, Rakhshan et al. found that education based on Leventhal perception improved the domains of lifestyle but did not affect the subscales of perception [16]. This raises the question of how it can improve lifestyle without improving perception. The reason for this difference could be that education focuses on lifestyle, not illness perception.

The results of this study can help healthcare providers to improve the healthy lifestyle of patients with hypertension by identifying the possible role of subscales of illness perception, including identity, acute/chronic timeline, personal control, treatment control, disease coherence, and cyclical timeline. Also, the use of Leventhal’s self-regulatory model in the educational curriculum of medical science students can be considered. Moreover, It is suggested that healthcare providers should design and implement the educational program based on the model to improve the lifestyle of patients with hypertension.

The present study helped to improve the illness perception, including subscales of identity, timeline acute/chronic, personal control, treatment control, illness coherence, and timeline cyclical, as well as the lifestyle of patients with hypertension. Moreover, the presence of an external academic observer and the active participation of patients in the research were considered the strengths of this study.

There were some limitations in carrying out the study, including the lack of a specific questionnaire for measuring the illness perception and lifestyle of patients with hypertension, the short duration of follow-up to determine possible long-lasting effects for maintaining a healthy lifestyle. However, the main limitation of the study was the lack of blinding of the study, mostly due to the nature of the intervention. Although the external academic supervisor supervised the research process, the findingsould be interpreted cautiously.

## Conclusion

The results of the present study showed that, the intervention based on Leventhal’s self-regulatory model brings about significant changes in the subscales of illness perception, except for consequences and emotional representations as the lifestyle of patients in the intervention group.

The replication of the current study with more intervention sessions, and longer follow-up period with study blinding is suggested for future research. It is also suggested to use other behavioral and spiritual interventions in similar research and evaluate their effects on the perception of consequences and emotional representations in patients with hypertension.

## Data Availability

The datasets used and/or analyzed during the current study are available from the corresponding author on reasonable request. However, all data generated or analyzed during this study are included in this published article.

## Abbreviations

HTN: Hypertension
DASH: Dietary approaches to stopping hypertension
LSQ: Lifestyle questionnaire
IPQ-R: Revised illness perception questionnaire
IP: Illness perception
